# Size distribution dependence of collective relaxation dynamics in a two-dimensional wet foam

**DOI:** 10.1038/s41598-021-82267-4

**Published:** 2021-02-02

**Authors:** Naoya Yanagisawa, Rei Kurita

**Affiliations:** Department of Physics, Tokyo Metropolitan University, 1-1 Minamioosawa, Hachiouji-shi, Tokyo, 192-0397 Japan

**Keywords:** Structure of solids and liquids, Phase transitions and critical phenomena, Soft materials

## Abstract

Foams can be ubiquitously observed in nature and in industrial products. Despite the relevance of their properties to deformation, fluidity, and collapse, all of which are essential for applications, there are few experimental studies of collective relaxation dynamics in a wet foam. Here, we directly observe how the relaxation dynamics changes with increasing liquid fraction in both monodisperse and polydisperse two-dimensional foams. As we increase the liquid fraction, we quantitatively characterize the slowing-down of the relaxation, and the increase of the correlation length. We also find two different relaxation modes which depend on the size distribution of the bubbles. It suggests that the bubbles which are simply near to each other play an important role in large rearrangements, not just those in direct contact. Finally, we confirm the generality of our experimental findings by a numerical simulation for the relaxation process of wet foams.

## Introduction

Foams constitute a soft jammed system, and have many unique mechanical properties. For example, they exhibit both elasticity and fluidity, making them distinct from ordinary fluids and solids. They are also widely seen in daily life, from foods and beverages to pharmaceuticals, cleaning products, cosmetics and building materials such as building insulation and flame-retardant barriers^1–14^. Foams can take three states depending on the liquid fraction $\phi$; these are called dry, wet and bubbly foams, from low to high $\phi$^13,14^. The dry state is made up of polyhedral bubbles and is more jammed than the wet state, which consists of round bubbles. Bubbly foams also consist of spherical bubbles, but do not have an elastic modulus under small shear^15^. Thus, although both dry and wet foams are jammed, bubbly foams are not: the transition from wet to bubbly foam is the jamming transition for this system. The transition point $\phi _{J}$ is located at $\phi _{J}$ $\sim$ 0.16 in two-dimensional foams and $\sim$ 0.36 in three-dimensional foams^16^.

There has been a surge of interest in studies of rearrangements in foams, due to their relation to macroscopic properties such as elasticity and fluidity. In a dry foam, the liquid films are sometimes rearranged and the it induces coarsening of bubbles over time^1,2^. It has also been reported that bubbles are simply stretched when a bubble collapses^13^. As the liquid fraction $\phi$ increases and the dry foam is transformed into a wet foam, the bubbles are rearranged more smoothly^14^, exhibiting enhanced fluidity. Recently, rearrangements and relaxation in jammed systems near the jamming point have excited significant interest. In the wet foam close to the jamming point, the bubbles are almost circular. In simulations using a soft particle model, where particles are allowed to overlap, it has been reported that the contact number per particle *Z* and rearrangements close to the jamming point exhibit critical behavior^17–19^. It has been also reported that a small perturbation to a particle in a soft jammed system leads to random motion in particles close to the perturbated particle and elastic deformation in particles far away; the threshold length dividing the regimes increase near the jamming transition^20^. In an experimental study, it was noted qualitatively that foam coarsening becomes slower near the jamming point^21^.

Yet, it should be noted that there are notably fewer in-situ observations of relaxation in jammed wet foams, particularly how the relaxation depends on the bubble size distribution. It is still unclear how structural relaxation occurs in the wet foams and what the key parameters are for relaxation. Thus, in this letter, we experimentally investigate the dynamical properties of a wet foam. We observe the dynamical response of two-dimensional foams by injecting a constant amount of solution into the foam. The slight increase in liquid fraction as a perturbation induces a rearrangement of bubbles. It is found that both the relaxation time and the correlation length associated with the rearrangement event increase as $\phi$ increases. We also find that the nature of the collective rearrangement depends on the size distribution of the bubbles. This suggests that mesoscopic structure, rather than bubbles in contact, plays an important role in large rearrangements. Furthermore, we perform the numerical simulation by using the soft particle model, and find that the motion of particles depends on the size distribution of the particles and the hexagonal order.

## Results and discussion

**Figure 1:**
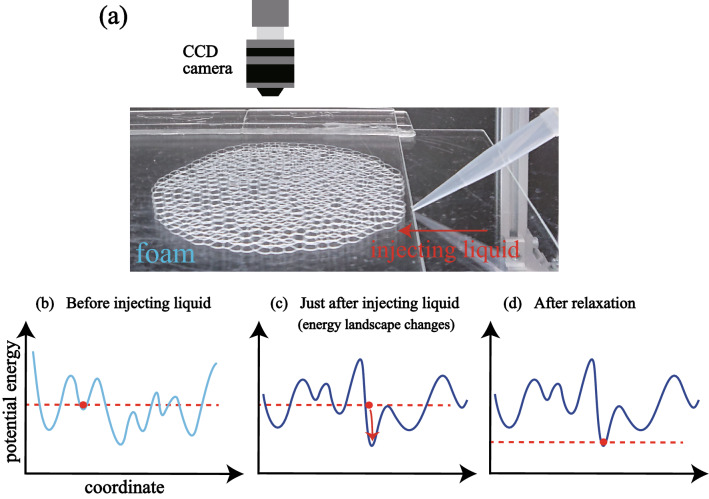
(**a**) Schematic of the experimental setup. We slowly inject a fixed amount of liquid (500 $\upmu$l) into a foam sandwiched between two glass plates from the outside using a micropipette. We then observe the relaxation from the top of the foam using a CCD camera. (**b**–**d**) Schematic of the energy landscape and the relaxation process. (**b**) Before injection, the foam is located in an energy basin. (**c**) Just after injection, the energy landscape changes since the number of possible states for the bubbles increases with increasing liquid fraction. We experimentally observe how the state relaxes into a new basin as shown in (**d**).

Firstly, we show the experimental setup (see Fig. 1a). We put a foam on a glass plate and cover it with another glass plate. The foam consists of the monolayer of bubbles and is considered quasi two-dimensional. In our experiment, we prepare two kinds of the foams, nominally ‘monodisperse’ and ‘polydisperse’. The mean diameter and area of the bubbles are 3.1 ± 0.3 mm and 7.7 ± 1.6 mm$^2$ for the monodisperse foam (polydispersity $\sigma$ = 0.107), and 4.4 ± 0.9 mm and 15.4 ± 6.8 mm$^{2}$ for the polydisperse foam ($\sigma$ = 0.201). We also note that the state of foams in our experiment is below jamming transition point $\phi _J \sim 0.16$, that is, the jammed state. See “Materials and methods” section for the details.

In order to study the relaxation of the foam, we apply a perturbation by injecting a constant amount of additional solution (500 $\upmu$l) from the outside using a micropipette, as shown in Fig. 1a. Figure 1b–d show schematic images for the energy landscape, which is usually related with the dynamics in jammed systems^22^. The red dot indicates the initial state before injection. Note that the state is in a basin; this is a steady state in the absence of any perturbation, where thermal energy can be neglected. By injecting liquid into the foam, the liquid fraction increases slightly and the energy landscape changes (Fig. 1c). The foam now relaxes into a new basin close to the initial state (Fig. 1d): this is the relaxation process we observe. We note here that the coarsening dynamics does not occur in the collective bubble relaxation in our experiment. We show the time evolution of the area *S* of a large bubble, a small bubble and the mean area of all bubbles in Supplementary Fig. S1. We randomly chose the large and the small bubbles. It is found that both *S* and the mean area are constant with respect to *t*.

**Figure 2:**
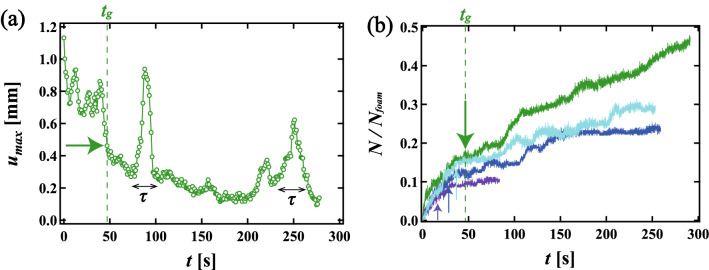
(**a**) The time evolution of the maximum of the displacement vector during the time interval $\Delta t$ = 10 s at $\phi _{2D} = 0.11$ in a monodisperse foam. *t* = 0 s is the time just after the solution is injected. The effect of the injection can be neglected after $t_g$, the green arrow in (**a**). Sharp peaks are found around 80 s and 250 s. These times correspond to large collective rearrangement events. $\tau$ is the interval from the start to the end of a single, discrete rearrangement event. (**b**) Cumulative number of rearranged bubbles $N/N_{foam}$ during the relaxation process as a function of time *t*, where $N_{foam}$ is the total number of bubbles. Each line corresponds to *N* at $\phi _{2D}$ = 0.110 (green), 0.093 (light blue), 0.061 (blue) and 0.042 (purple). Arrows correspond to $t_g$ as shown in (**a**).

Here, we consider key parameters to characterize the dynamics of the relaxation process. Immediately after injection, the bubbles near the injection point flow and subsequently return to their original positions. This is followed by several large collective rearrangements (see Supplementary Movie 1). In order to estimate the effect of the injection, we compute the displacement vector of bubbles *i* $u_i (t, \Delta t)$, where $\Delta t$ is a time interval. In order to obtain sufficient accurate velocity, the displacement of the bubble should be much larger than the spatial resolution (0.238 mm). Thus we chose $\Delta t$ = 10 s for $\phi _{2D}$ > 0.07 while $\Delta t$ = 1 s for $\phi _{2D}$ < 0.07 since the velocity decreases with increasing $\phi _{2D}$. Those time scales are shorter than the relaxation time of the single collective rearrangement event $\tau$. We checked that our results are not changed in a condition that $\Delta t$ is shorter than $\tau$. We firstly consider the maximum of the displacement vectors $u_{max}$. Figure 2a shows the time evolution of $u_{max}$ for monodisperse foams. $u_{max}$ greatly decreases just after the injection; this corresponds to the bubbles returning. $u_{max}$ decreases gradually since the system is slowly aging after a time $t_g$ shown by the green arrow in Fig. 2a. Here, we confirm that the injected volume and the injection speed both affect $t_g$, but do not affect the dynamics of the foam after $t_g$ i.e. the dynamics after $t_g$ only corresponds to the relaxation dynamics illustrated in Fig. 1b–d. Note that the relaxation dynamics we discuss below is all after $t_{g}$. We also find sharp increases in $u_{max}$ around $t =$ 80 s. From direct observation of the foam over time (see Supplementary Movie 1), we confirm that these correspond to discrete, significant collective rearrangements of the bubbles. Here, we define a relaxation time $\tau$ as the time from the start of the peak to the end, where $u_{max}$ is deviated from the base line. Thus $\tau$ is associated with a single collective event. We also confirm that the relaxation time using $\Delta t$ is almost same as $\tau$ determined by in-situ observations. An example of $\tau$ at $\phi _{2D}$ = 0.11 is presented in Fig. 2a. We also find another peak at *t* = 250 s, where two peaks are overlapped. In this case, we also checked the spatial distribution of the rearranged bubbles in order to distinguish some collective relaxation events. The latter peak corresponds to the collective rearrangement event, while the former peak is just a T1 event. Thus we only chose the width of the latter peak as $\tau$. This phenomenon corresponds to an avalanche, where one rearrangement induces another in a different location. If we can separate the events in the avalanche through direct observation, we measure $\tau$ as the relaxation time of each collective event, while we excluded the data when two collective rearrangements are temporally and spatially merged and they are undistinguished. We note that it is difficult to distinguish individual rearrangements near $\phi _{J}$. We also note that the same behavior is seen for polydisperse foams.

We also count the number of bubbles which undergo a rearrangement during the relaxation process as a function of time. We say that a bubble is “rearranged” if its contact bubble changes. Figure 2b shows the cumulative number of rearranged bubbles *N* as a function of time *t*. We set *t* = 0 s to be just after the injection; *N* is normalized by $N_{foam}$, where $N_{foam}$ is the total number of bubbles in the foam. The green, light blue, blue, and purple lines correspond to *N* at $\phi _{2D}$ = 0.110, 0.093, 0.061 and 0.042, respectively. Each arrow in Fig. 2b indicates $t_g$, when the local liquid fraction in the foam becomes approximately constant, that is, when the effect of the injection almost disappears. The rapid increase in *N* before $t_g$ is consistent with the decrease in $u_{max}$ shown in Fig. 2a. We see that for lower $\phi _{2D}$, *N* is small and saturates at an early stage, whereas for higher $\phi _{2D}$, *N* starts larger and continues to increase gradually for longer.

**Figure 3:**
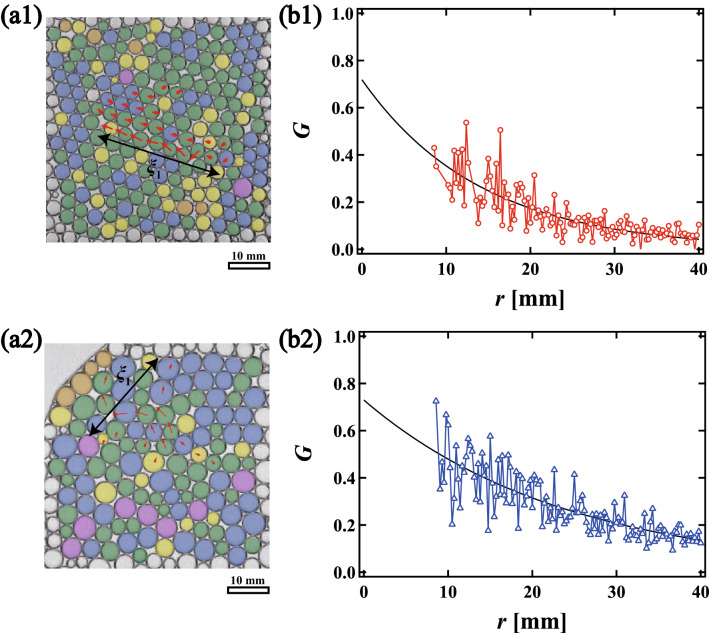
Displacement vector fields of rearranged bubbles in (**a1**) a monodisperse foam when $\phi _{2D} = 0.11$ from 119 s to 278 s and (**a2**) a polydisperse foam when $\phi _{2D}$ = 0.093 from 66 s to 146 s. Vectors are shown three times their actual lengths for visibility. (**b1**) Displacement correlation function *G* as a function of distance *r* for the monodisperse foam. (**b2**) *G* for the polydisperse foam. The black solid line is an exponential fitting by $\exp (r/\xi )$. Correlation length (size) $\xi _{1}$ is defined as the greatest distance between bubbles in a single collective rearrangement event. We also define $\xi _2$ as the exponential decay length of *G*.

**Figure 4:**
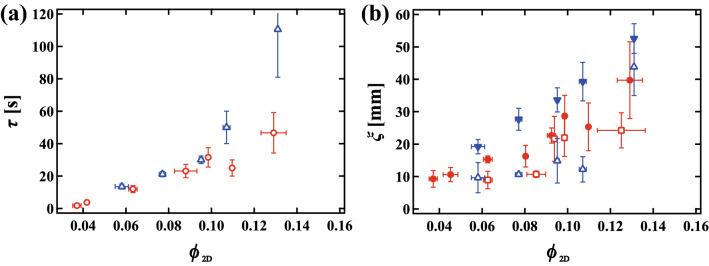
(**a**) The mean relaxation time $\tau$ of a single collective rearrangement event averaged over several collective rearrangements as a function of $\phi _{2D}$. Red circles and blue triangles indicate $\tau$ in monodisperse and polydisperse foams, respectively. (**b**) The mean correlation length $\xi$ of a single collective rearrangement as a function of $\phi _{2D}$. Red filled circles and red open squares indicate $\xi _{1}$ and $\xi _{2}$ in the monodisperse foams, respectively. Blue filled reverse triangles and open triangles indicate $\xi _{1}$ and $\xi _2$ in the polydisperse foam. The error bars correspond to the distributions of the data.

Next, we consider how to associate the correlation length with the collective motion. Figure 3a1 shows displacement vectors for collectively rearranged bubbles in the monodisperse foam from *t* = 119 to 278 s at $\phi _{2D}$ = 0.11. The vectors are shown three times their actual lengths for visibility. Note that these displacements are over a single, collective rearrangement event, and that collectively rearranged bubbles are those which change their contact bubbles over the same time interval. We find that in monodisperse foams, collective rearrangement occurs through slip-like displacements. A similar analysis may be applied to a polydisperse foam. Figure 3a2 shows displacement vectors of bubbles in a polydisperse foam from *t* = 66 to 146 s when $\phi _{2D}$ = 0.093 (see Supplementary Movie 2). A random rearrangement occurs which is distinct from the sliding motion seen in monodisperse foams. This random motion is consistent with observations in the soft binary particle simulations^20^.

Here, we define two correlation lengths for each collective rearrangement event. $\xi _{1}$ is defined as the distance between the two collectively rearranged bubbles which are furthest apart, as shown in Fig. 3a1. We obtain the displacement using a single collective rearrangement event. The other correlation length is defined by a displacement correlation function $G(r) = F(r)/F(0)$, where *F*(*r*) is

1 $$\begin{aligned} F\left( r\right) =\frac{1}{N_j}\displaystyle \sum _{i,j}|\vec {u_i}||\vec {u_j}|. \end{aligned}$$

For a collectively rearranged bubble *i*, $\vec {u_i}$ is the displacement vector during a single collective rearrangement, while $\vec {u_j}$ is taken over all $N_j$ bubbles *j* at a distance *r* from bubble *i*. Figure 3b1,b2 show *G*(*r*) as a function of *r* for the data shown in Fig. 3a1,a2, respectively. We obtain the correlation length $\xi _{2}$ (the black line) by the exponential fitting $G=\text{exp}(-r/\xi )$.

We are now ready to quantitatively study dynamical behavior in a wet foam. Figure 4a shows the mean relaxation time $\tau$ averaged over several (> 5) collective events as a function of $\phi _{2D}$ in monodisperse (circle) and polydisperse (triangle) foams. Similarly, Figure 4b shows the mean correlation lengths averaged over several (> 5) collective events as a function of $\phi _{2D}$. Red filled circles and blue filled reverse triangles in Fig. 4b show $\xi _{1}$ for the monodisperse foam and for the polydisperse foam, respectively. Red open squares and blue open triangles show $\xi _{2}$ for the monodisperse foam and for the polydisperse foam, respectively. Note here that the distribution of $\tau$ and $\xi$ are plotted as the error bar in Fig. 4a,b, respectively. Although the nature of the rearrangements is different for monodisperse and the polydisperse foams, as discussed below, $\tau$ and $\xi$ similarly increase with increasing $\phi _{2D}$.

**Figure 5:**
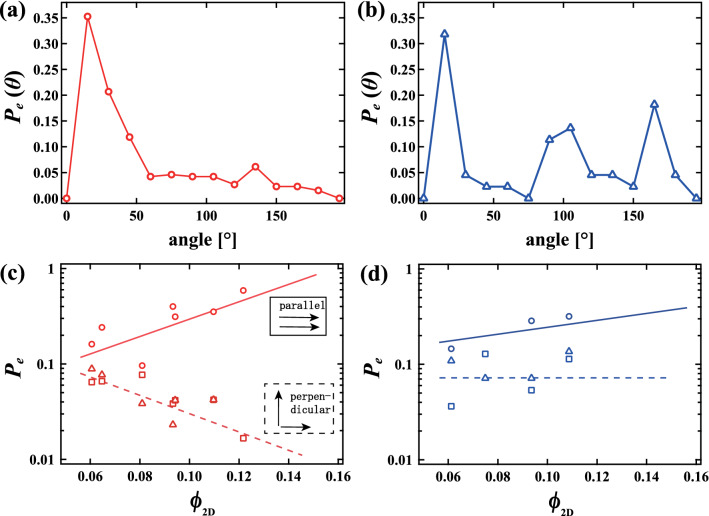
Probability distribution $P_{e}( \theta )$ of the angle between displacement vectors of bubbles in contact in collective rearrangement events at (**a**) $\phi _{2D}$ = 0.11 in a monodisperse foam and (**b**) $\phi _{2D}$ = 0.11 in a polydisperse foam. $P_{e}( \theta )$ has only one peak near $\theta = 0$ for the monodisperse foam, while $P_{e}( \theta )$ has another peak around $\theta$ = 90° for the polydisperse foam. The probability of parallel and perpendicular displacements as a function of $\phi _{2D}$ in (**c**) monodisperse and (**d**) polydisperse foams. Circles, squares and triangles indicate probabilities that the angles are 0$^{\circ }$ to 15$^{\circ }$, 75$^{\circ }$ to 90$^{\circ }$, and 90$^{\circ }$ to 105$^{\circ }$, respectively. The solid lines and dashed lines represent slip-like parallel displacements and perpendicular displacements, respectively. Slip-like parallel motion is more likely to be seen closer to the jamming point in monodisperse foams, but is independent of proximity to the jamming point in polydisperse foams.

We may also compute how collective the motion of the bubbles is for a single rearrangement event. We calculate the probability distribution $P_{e}({\theta })$ for the angle between displacement vectors of bubbles in contact participating in a rearrangement event. Figure 5a,b show $P_{e}( \theta )$ at $\phi _{2D}$ = 0.11 for the monodisperse and polydisperse foams, respectively. $P_{e}( \theta )$ has only one peak near $\theta = 0$ for the monodisperse foam, while there is another peak around $\theta$ = 90$^\circ$ for the polydisperse foam. Figure 5c shows $P_\parallel$ (circle), $P_{\perp 1}$ (square) and $P_{\perp 2}$ (triangle) as a function of $\phi _{2D}$ in the monodisperse foam, where $P_\parallel$, $P_{\perp 1}$ and $P_{\perp 2}$ are the probabilities that the angle is between 0$^\circ$ and 15$^\circ$, 75$^\circ$ and 90$^\circ$, and 90$^\circ$ and 105$^\circ$, respectively. As $\phi _{2D}$ increases, we find that $P_\parallel$ increases while $P_\perp$ decreases. This indicates that bubbles are more likely to move in the same direction near the jamming point in the monodisperse foam. Figure 5d shows $P_\parallel$ (circle), $P_{\perp 1}$ (square) and $P_{\perp 2}$ (triangle) as a function of $\phi _{2D}$ in the polydisperse foam. Interestingly, we see that $P_\parallel$ and $P_\perp$ remain almost constant as $\phi _{2D}$ increases. This shows that bubbles move just as randomly, regardless of liquid fraction. Thus, the rearrangement dynamics depends on the size distribution of the bubbles, both qualitatively and quantitatively.

We consider the structural difference between monodisperse and polydisperse foams. Firstly, we compute the nominal contact number $Z^{*}$ for each bubble. We define nominal contact as when the gap between the bubble interfaces is below 0.238 mm, the spatial resolution of our observation method. Thus, $Z^{*}$ may be larger than the actual contact number *Z*. Note that the foam adopts a disordered configuration as shown in Fig. 3a1 for the monodisperse foam and (a2) for the polydisperse foam. Different colors indicate $Z^{*}$ of each bubble; orange, yellow, green, blue and purple correspond to bubbles with 3, 4, 5, 6 and 7 contacts, respectively. We find $Z^{*}$ = 5.34 at $\phi _{2D}$ = 0.094 for the monodisperse foam and $Z^{*}$ = 5.33 at $\phi _{2D}$ = 0.093 for the polydisperse foam. Thus, there is very little structural difference when we look at the contact number. However, if we use a nearest neighbor method of associating bubbles rather than contact, we begin to see hexagonal order in the monodisperse foam, but not in the polydisperse foam. We define a bubble *k* as a nearest neighbor if $r_{ik} < 1.3 d_{i}$, where $r_{ik}$ is the distance between *i* and *k* and $d_{i}$ is the diameter of bubble *i*. We use the local hexatic order parameter $\psi ^i_{6} = \frac{1}{n_i} |\Sigma _{k} \exp (j 6 \theta _k)|$, where $n_i$ is the number of nearest neighbors, *j* is the imaginary unit, and $\theta _k$ is the angle of the relative vector $\vec {r_k} - \vec {r_i}$ with respect to *x* axis. We may also compute $\langle \psi _6 \rangle$, which is the particle average of $\psi ^i_{6}$. We obtain $\langle \psi _6 \rangle$ = 0.772 at $\phi _{2D}$ = 0.094 for the monodisperse foam and 0.587 at $\phi _{2D}$ = 0.093 for the polydisperse foam. We also consider the proportion $\alpha$ of hexatic order bubbles, where $\psi ^i_{6} > 0.85$ . We obtain $\alpha$ = 0.542 for the monodisperse foam and 0.267 for the polydisperse foam. Furthermore, we investigate $\psi ^i_{6}$ by using a Voronoi tessellation as well as a separation cutoff. As a result, we obtain $\langle \psi _6 \rangle$ = 0.822 at $\phi _{2D}$ = 0.094 for the monodisperse foam and 0.545 at $\phi _{2D}$ = 0.093 for the polydisperse foam in the experiment. We find that both $\langle \psi _6 \rangle$ (cut off and Voronoi tessellation) are almost the same. Thus, the slip-like motion seen during rearrangements may be related to this hexagonal order. Here, we note that the predictions from numerical simulations is limited to small perturbations, that is, displacements in the linear relaxation regime^23,24^; the rearrangements we see in our experiment are large and non-linear. Our results suggest that it is not only bubbles in contact, but those which are simply near to each other which play an important role in large, non-linear rearrangements.

We also note that rearrangements sometimes do not occur after liquid injection. This reason may be that the energy landscape is not changed. This can occur when the system size is not large enough. If the size of the foam is infinite, we may assume that collective rearrangements will always occur. Finite size effects may also play a role in the relaxation of the foam in our experiment near the jamming point, which may affect the relaxation dynamics. In the future, we will investigate relaxation dynamics using larger foams to further enhance accuracy near $\phi _{J}$ and expand upon our discussion.

**Figure 6:**
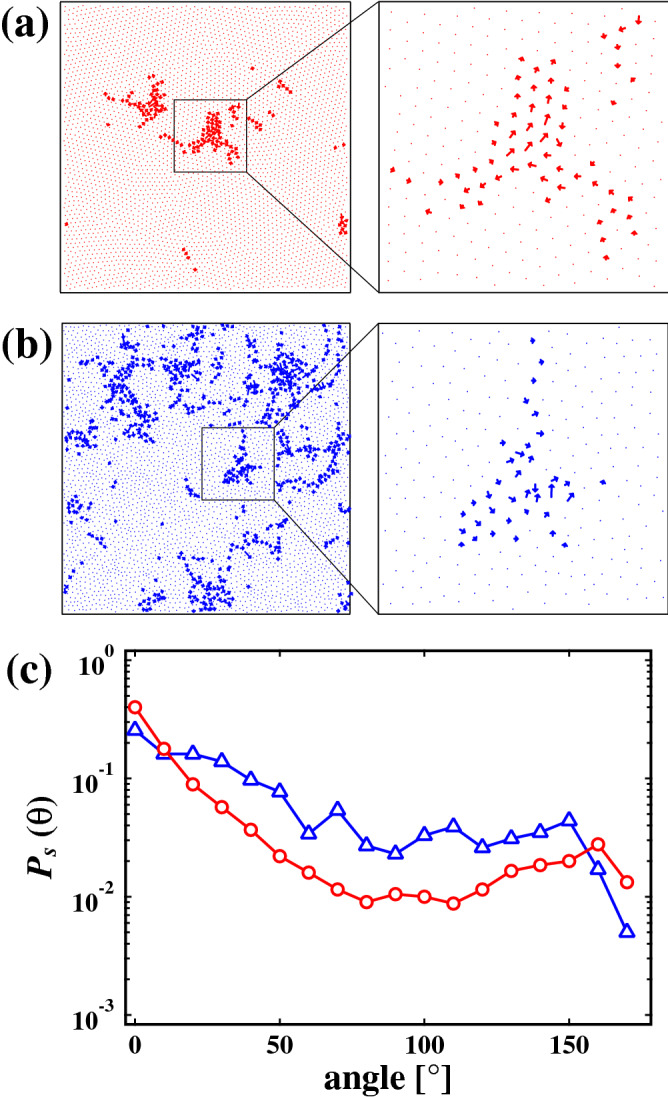
Displacement vector field of particles with (**a**) $\sigma$ = 0.10 and (**b**) $\sigma$ = 0.20 over the time interval $4.0 \times 10^{4}$ to $1.0 \times 10^{5}$ and $2.5 \times 10^{4}$ to $1.0 \times 10^{5}$, respectively. We change $\phi _v$ from 0.114 to 0.125 for $\sigma$ = 0.10 and from 0.116 to 0.128 for $\sigma$ = 0.20. The left images are zoomed in on a collective motion regions for the simulation systems. Vectors are shown the ten times larger than their actual lengths for visibility. (**c**) Probability distribution $P_{s}(\theta )$ for the angle between displacement vectors of particles in contact each other. Red circle and blue triangle symbols indicate particles with $\sigma$ = 0.10 and $\sigma$ = 0.20, respectively.

In the experiments, many experimental factors, such as the concentration of the surfactant, viscosity, deformation, elastic modulus, type of the surfactant and so on, can be related with the relaxation. Thus, we perform numerical studies of the relaxation dynamics to confirm the generality of our experimental results. In wet foams the soft particle model is one of the simplest model for investing the relaxation process^19^. In order to prepare the initial state, the particles are placed in the simulation box, and we repeat increasing the size of all particles by 1% and relaxing the system until the void fraction $\phi _v$ becomes about 0.11. We note that $\phi _v$ corresponds to $\phi$ in the experiment. Then we decrease the size of all particles by 1% at time $t = 0$. This shrink of the particles corresponds to the injection of liquid in our experiment. Figure 6a,b are the displacement vector field of particles with $\sigma$ = 0.10 and $\sigma$ = 0.20 over the time interval $4.0 \times 10^{4}$ to $1.0 \times 10^{5}$ and $2.5 \times 10^{4}$ to $1.0 \times 10^{5}$, respectively. We change $\phi _v$ from 0.114 to 0.125 for $\sigma$ = 0.10 and from 0.116 to 0.128 for $\sigma$ = 0.20. It seems that slip-like displacements occur for $\sigma$ = 0.10, whereas random-like displacements occurs for $\sigma$ = 0.20. Figure 6c shows the probability distribution $P_{s}(\theta )$ for the angle between displacement vectors of particles in contact each other, and red circle and bule triangle symbols indicate particles with $\sigma$ = 0.10 and $\sigma$ = 0.20, respectively. It is found that $P_{s}(\theta )$ obtained from the simulation is almost consistent with $P_{e}(\theta )$ obtained from the experiment. Moreover, we investigate *Z* and $\psi ^i_{6}$, and obtain $Z = 4.465$ and $\langle \psi _6 \rangle = 0.855$ for particles with $\sigma$ = 0.10, and obtain $Z=4.471$ and $\langle \psi _6 \rangle = 0.556$ for particles with $\sigma$ = 0.20. We also find that in a Voronoi tessellation $\langle \psi _6 \rangle$ = 0.818 and 0.522 for $\sigma$ = 0.10 and $\sigma$ = 0.20, respectively. Thus, we establish that the slip-like motion seen during the relaxation process is related to the hexagonal order. Our simulation results also show that the relaxation dynamics of the jammed wet foams close to the jamming point is universal, that is, it does not depend on the experimental factors such as deformation, the type of the surfactant, its concentration and so on.

To summarize, we experimentally investigated the relaxation dynamics of wet foams. By injecting a constant amount of liquid into a two-dimensional foam, the energy landscape of the foam was changed and a relaxation process was observed. We saw that the relaxation time and correlation lengths associated with discrete relaxation events both increase with $\phi$ for both monodisperse and polydisperse foams. Furthermore, we found that the way in which the rearrangements occurred depended on the size distribution of the bubbles. A slip-like motion was observed in monodisperse foams, while a more random motion was seen in polydisperse foams. We found that the hexagonal order found from nearest neighbor bubbles, not only bubbles in contact, may be crucial for the difference in the motion. We also verified these experimental results by performing the numerical simulation of the relaxation process of the wet foam using the soft particle model. In the future, we hope to study the transition from slip-like to random motion in more depth. We believe these results, as well as those planned, form key experimental investigations which will significantly advance our understanding of not only foams but also of soft jammed systems, and may guide the development of new applications for soft condensed matter.

## Materials and methods

We use an aqueous solution of household detergent (CHAMY, Lion Co., Japan), diluted to 25% with deionized water. The foams are created using a capillary glass tube connected to an air pump. In this generation method, it is well known that the generated bubble size depends on the nozzle size and gas flow rates^25^. In this study, we calibrate the bubble size distribution by controlling the air-injection speed. We create monodisperse and polydisperse foams by using the speed between approximately 10 and 70 ml/min. The diameter of nozzle is 0.7 mm. We define the bubble size dispersity as

2 $$\begin{aligned} \sigma = \frac{\sqrt{\frac{1}{N_{foam}}\sum _{i}\left( \langle {d}\rangle -d_{i}\right) ^{2}}}{\langle {d}\rangle }, \end{aligned}$$

where $\langle {d}\rangle$ and $d_{i}$ are mean diameter of bubbles and the diameter of bubble *i*, respectively. We obtain $\sigma$ = 0.107 for monodisperse foams and 0.201 for polydisperse foams. The sample thickness is set to 2.1 mm using a spacer. The number of bubbles is 350 $\sim$ 500. We take videos of the relaxation process after liquid injection using a CCD camera (KEYENCE, VW-9000) at 30 frames per second. We examine static and dynamical properties using an image analysis technique developed in-house. Spatial resolution (0.238 mm) was exchanged for access to a larger field of view over time. We also compute a two-dimensional liquid fraction $\phi _{2D}$ using image analysis, which corresponds to the two-dimensional liquid fraction in a cross section through the center of the sample. $\phi _{2D} = 1 - S_{bubble}/S_{foam}$, where $S_{bubble}$ is the area of the bubble region, and $S_{foam}$ is the whole area of the foam^14^. Note that the upper limit of $\phi _{2D}$ in our experiment is about 0.13 since the relaxation time becomes much longer near $\phi _{J}$, and the influence of evaporation and drainage is not negligible. We also note that the state of foams in our experiment is below jamming transition point $\phi _J \sim 0.16$, that is, the jammed state.

We use the soft particle model for investigating the relaxation process of the wet foam. This particle is frictionless circular disk and the distribution of the particle sizes is a Gaussian function. Pairs of the disks *i* and *j* interact via the pairwise harmonic repulsive potential:

3 $$\begin{aligned} U\left( r_{ij}\right) =\frac{\epsilon }{2}\left( 1-\frac{r_{ij}}{D_{ij}}\right) ^{2}\Theta \left( D_{ij}-r_{ij}\right) , \end{aligned}$$

where $\epsilon \left( =1\right)$ is the characteristic energy scale of the interaction, $D_{ij}=(R_{i}+R_{j})/2$, $r_{ij}$ is the center-to-center distance between disks *i* and *j*, and $\Theta$ is the Heaviside function. In this model, the overlap between pairs of particles is allowed. We take 4000 particles with $\sigma$ = 0.10 and $\sigma$ = 0.20, which correspond to monodisperse and polydisperse foams used in our experiment, respectively. The periodic boundary conditions are applied in the simulation box.

## Supplementary information

Supplementary Information.

Supplementary Movie 1.

Supplementary Movie 2.
