# Supplementary material for: Size distribution dependence of collective relaxation dynamics in a two-dimensional wet foam
Source: Sci Rep. 2021 Feb 2;11:2786. doi: 10.1038/s41598-021-82267-4 (PMC7854744; doi:10.1038/s41598-021-82267-4)
Supplement: Supplementary file 1 — Supplementary Information. [file 41598_2021_82267_MOESM1_ESM.pdf]

Supplementary Information for
"Size distribution dependence of collective relaxation dynamics in a two-dimensional wet foam"

Naoya Yanagisawa and Rei Kurita
Department of Physics, Tokyo Metropolitan University, Tokyo 192-0397, Japan

(Supplementary Movie 1) The movie of the relaxation process for φ_{2D} = 0.11 in a monodisperse foam.

We took this movie by using a CCD camera. This movie is at 50x speed.

(Supplementary Movie 2) The movie of the relaxation process for φ_{2D} = 0.093 in a polydisperse foam.

We took this movie by using a CCD camera. This movie is at 30x speed.

Time evolution of the area of bubbles

Figure S1 shows the area of a large bubble (blue), a small bubble (red), and the mean area of all bubbles during the relaxation process as a function of time *t*. We randomly chose the large and the small bubbles. It is found that both *S* and the mean area are constant with respect to *t*. Thus, we consider that the coarsening dynamics does not occur in the collective bubble relaxation in our experiment.

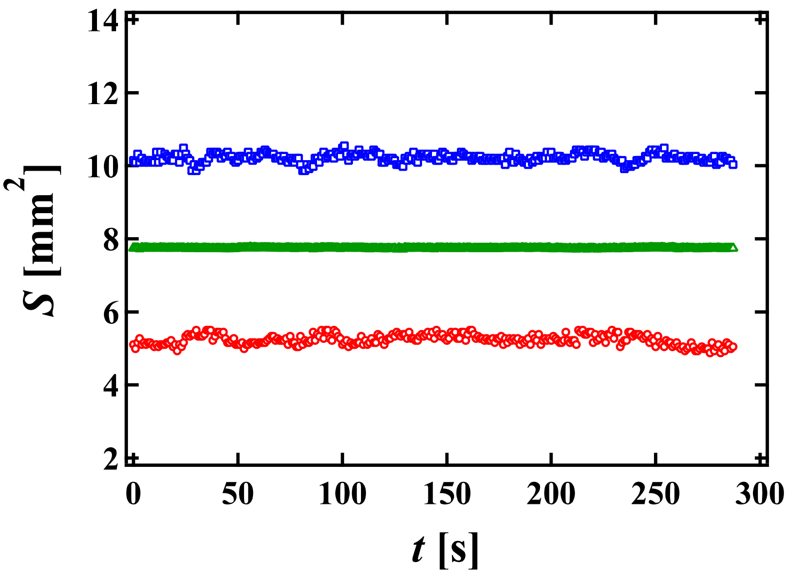

Fig.S1. The area of bubbles *S* during the relaxation process as a function of time *t* at φ_{2D} = 0.11 in a monodisperse foam. Blue square and red circle symbols indicate the area of a large and that of a small bubble, respectively. Green triangle symbols indicate the mean area of all bubbles.
